# Spectroscopy of Deep Traps in Cu_2_S-CdS Junction Structures

**DOI:** 10.3390/ma5122597

**Published:** 2012-12-04

**Authors:** Eugenijus Gaubas, Ievgen Brytavskyi, Tomas Ceponis, Vidmantas Kalendra, Audrius Tekorius

**Affiliations:** 1 Institute of Applied Research, Vilnius University, Sauletekio Ave. 9-III, Vilnius 10222, Lithuania; E-Mails: tomas.ceponis@ff.vu.lt (T.C.); vidmantas.kalendra@ff.vu.lt (V.K.); audrius.tekorius@ff.stud.vu.lt (A.T.); 2 Odessa I.I. Mechnikov National University, Dvoryanskaya str. 2, Odessa 65082, Ukraine; E-Mail: brytav@ukr.net

**Keywords:** Cu_2_S-CdS heterojunctions, CdS polycrystalline layers, carrier traps, optical injection DLTS, photo-ionization spectroscopy

## Abstract

Cu_2_S-CdS junctions of the polycrystalline material layers have been examined by combining the capacitance deep level transient spectroscopy technique together with white LED light additional illumination (C-DLTS-WL) and the photo-ionization spectroscopy (PIS) implemented by the photocurrent probing. Three types of junction structures, separated by using the barrier capacitance characteristics of the junctions and correlated with XRD distinguished precipitates of the polycrystalline layers, exhibit different deep trap spectra within CdS substrates.

## 1. Introduction

CdS and Cu_2_S compounds are of great interest owing to their unique properties in variation of the stoichiometric composition, due to nanocrystal morphology, valence states and their potential applications in numerous fields [1,2]. Copper sulfide (Cu_2_S), as a p-type semiconductor, has recently attracted considerable scientific and technological interest as a promising material in applications of solar cells [3,4]. Cadmium sulfide CdS is also a widely used material with many advanced technological applications—much research interest comprises the possible applications of CdS in fabrication of various optoelectronic devices [5] based on CdS polycrystalline films, which are obtained by various methods. Several techniques, such as solid state reactions (dry processing) [6], vacuum evaporation [7], sputtering [8], spray pyrolysis [9] and sulfurization of Cu films [10] have been applied for the production of Cu_2_S films on CdS substrate. Particularly, Cu_2_S-CdS heterojunction nanostructures were used for obtaining core–shell nanowire devices [11] with photovoltaic applications. These nanowire photovoltaic cells exhibit relative stability, enabling excellent charge separation with minimal minority carrier recombination. Thus, spectroscopic data on polycrystalline CdS layers is desirable to predict optoelectronic and radiative features of these materials.

In this work, the polycrystalline films containing heterostructures formed by dry deposition method [12] have been investigated. The heterostructures were formed by employing a substitution technique when a layer of copper sulfide is formed directly on the substrate layer of CdS during heat treatment using a pre-printed copper chloride film formed by evaporation in vacuum. This technology is promising due to low cost, easy processibility, the possibility of large area fabrication together with satisfactory structure quality. However, such a heterojunction made of p-type Cu_2_S and n-type CdS is a rather complicated system because of the interface between two materials with different electron affinities, band gaps and polycrystalline structure. The lattice mismatch and inter-diffusion of components cause defects at or near the interface that strongly affect the junction properties. In particular, there appears to be a Cu diffusion into the CdS layer adjacent to the interface, which leads to a stoichiometry changing of Cu*_x_*S layer and may cause a shunting effect within the CdS layer [13]. Due to polycrystalline material and the lattice mismatch between the material components, a large density of trapping and recombination centers appears. These disordered material areas cause the effect of photo-induced modulation of the junction potential barrier. In this work, to evaluate an impact of traps on parameters of substrate CdS layers, spectra of deep traps have been examined by combining the capacitance deep level transient spectroscopy technique together with white LED light additional illumination (C-DLTS-WL) and the photo-ionization spectroscopy (PIS) implemented by probing of the DC photocurrent changes [14].

## 2. Samples of the Polycrystalline CdS-Cu_2_S Layered Structures

The layered structures were produced together with a rear electrode. The back electrode was obtained by deposition of a transparent 100 nm thick SnO_2_ layer on glass substrate in vacuum. The CdS layer was deposited by evaporation in vacuum of pure CdS powder. The deposition rate was about of 0.3 μm/min, and a glass substrate was held at about 200 °C during deposition. A thin CdS layer of thickness less than 10 μm was not sufficient to prevent the shunting problem. So, the evaporation time was varied from 60 to 80 min to get a thick enough (15–26 μm) CdS layer. Enhancement of the CdS layer thickness leads to an unacceptable increase of the base region resistivity, thereby, a 20–24 μm thick CdS region was found to be optimal. Thermal anneal in vacuum at *T* = 450 °C for 30 min was exploited after deposition of CdS base region to perform the re-crystallization of the as-deposited CdS layer. Thermal treatment induces an increase of the crystallite size and a reduction of the crystal defects density [15]. These CdS substrate layers were examined by photo-ionization spectroscopy (PIS). For these measurements, a top electrode was made as a pressed metallic contact.

The top-layer of the Cu_2_S-CdS junction structure was formed directly from the base layer of CdS [12] during heat treatment with a pre-printed copper chloride film. There, a thin layer of CuCl (up to 1 μm thick) was evaporated onto the CdS film. The source and substrate temperature were kept at 600 °C and 250 °C, respectively. Then, samples were heated at 200 °C for 4 min in a vacuum chamber. The processed films were washed in distilled water to remove the CdCl_2_ layer formed in the reaction. The thickness of Cu_2_S layer is manipulated by varying of CuCl deposition time. The substitution reaction duration was fixed for 4 min, during which all the CuCl layer was reacted. The thickness of the Cu_2_S layer is determined by the initial thickness of the CuCl source-layer. Thus, the employed regimes, varying the CuCl evaporation time from 4 to 12 min, enabled us to change the thickness of Cu_2_S layer in the range from 200 to 1000 nm. These junction structures were employed for capacitance deep level transient spectroscopy (C-DLTS). As most of the Cu_2_S-CdS junction structures exhibited full depletion even at the smallest reverse voltages due to a lack of free carriers, the routine DLTS measurements were performed at steady-state bias illumination using white LED light (C-DLTS-WL).

## 3. Measurement Techniques and Regimes

**Figure 1:**
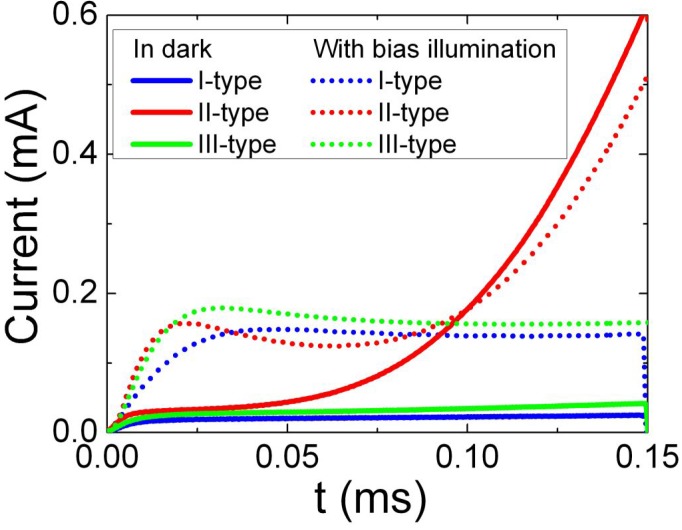
The typical barrier evaluation by linearly increasing voltage (BELIV) transients observed in structures of a different type Cu_2_S-CdS junction without (solid curves) and with steady-state wide spectral band illumination (dot curves) by applying the linearly increasing voltage pulse with 1.2 V amplitude at reverse polarity.

The barrier evaluation by linearly increasing voltage (BELIV) pulsed technique [16,17] was applied to separate the junction structures by the controlling of free carrier densities and generation currents within the CdS base region. Three types of samples had been distinguished by using the barrier capacitance characteristics of the junctions. Steady-state illumination determines photo-ionization of deep traps and, thus, an enhancement of free carrier density in the neutral region. This leads to an increase of barrier capacitance value due to enhancement of effective doping *C*_b0_ (*N*_Def_) = (*εε*_0_*S*^2^*eN*_Def_/2*U*_bi_)^1/2^~*N*_Def_^1/2^ (*N*_Def_ = *N*_D_ + *N*_T_*^+^* with donors *N*_D_ and ionized traps *N*_T_*^+^* densities, respectively). This result qualitatively correlates with changes of Cu_2_S-CdS junction characteristics under illumination, published in [13]. In our experiments, changes of barrier capacitance were obtained to be different (Figure 1) for the separated types of samples. In type-I samples, barrier capacitance is nearly independent of voltage, and these junctions behave like a capacitor due to a lack of free carriers. For steady illuminated samples of type-II and type-III, barrier capacitance increases relative to that value measured in the dark. This observation indicates a recovery of the junction under steady-state illumination.

Substrate temperature and layer deposition duration were the main parameters varied during the heterojunction formation process. The thickness of the base layer defines its resistivity and the size of the formed microcrystals. Samples with the thicker base CdS layer showed capacitance transients specific for type-I samples. The separated types of samples (characterized by inherent BELIV transients) also correlate with Cu*_x_*S layer thickness. The BELIV characteristics are close to those inherent for the type-II samples containing the thicker Cu*_x_*S layer. The revealed differences in doping and trap densities leading to significant variations of electrical characteristics of the junctions are caused by layer deposition regimes influencing the layer thickness, composition and structure.

The stoichiometric defects over Cu_2_S and CdS layers were resolved within XRD patterns. The XRD study indicated that type-II samples contain a larger amount of Cu*_x_*S phase precipitates than type-III samples. Type-II samples were fabricated at the longest deposition time *t*_dep_ and the highest temperature *T*_sb_.

In this work, the spectral measurements have been performed for different types of junction structures to identify specific deep traps according to published signatures. The C-DLTS measurements were performed by using a commercial spectrometer SemiTrap DLS-82E manufactured by SemiLab, Hungary. The sample was mounted within a liquid nitrogen immersion cryo-chamber. The correctness of the mounting circuit and a state of electrodes was controlled by C-V measurements. Also, C-V results obtained in the dark and with bias illumination are routinely employed for evaluation of the barrier capacitance and of the densities of traps. As revealed from BELIV characteristics, most of the Cu_2_S-CdS junction structures exhibited a full depletion condition even at the smallest reverse voltages. Then, a routine C-DLTS instrument and measurement regimes are non-applicable. Therefore, a steady-state bias illumination using white light LED with an emission wavelength in the range of 400–700 nm, mounted inside the cryo-chamber, has been employed. Thereby, the capacitance deep level transient technique is modified by using an optically induced conductivity regime [18], *i.e.*, the C-DLTS-WL technique is implemented in our measurements. Measurements of deep trap spectra have been implemented by C-DLTS-WL on CdS-Cu_2_S junction structures for the probing frequency range of 0.1–2.5 kHz. The base region of the junction (*i.e.*, CdS layer) is then examined. For separation of the overlapping DLTS peaks, the approximation of mono-exponential peaks close to Gaussian shape has been used, like that installed within the commercial software of the DLS-82E spectrometer. The shift of simulated overlapping peaks by varying probing frequency is reproduced right enough to identify traps, as published in the literature. This approach had been verified on Si junctions when DLTS spectra are complicated due to the simultaneous action of several known traps.

To verify the spectral structure of deep traps and to search peculiarities of thermally (C-DLTS-WL) and optically induced carrier transitions, the single layer (CdS) and samples with junction structures were investigated by using the photo-ionization spectroscopy (PIS) technique, where either DC photo-current or barrier capacitance charging current is probed. The photo-ionization spectra are recorded by using the 150 W halogen lamp light source, and the spectrum is dispersed by double-way monochromator. A sample is usually mounted in liquid nitrogen cryostat to reduce a leakage current.

The photo-ionization spectra have been analyzed by using a δ-potential deep center approach, known as the Lucovsky model [19]. The red-threshold of the photo-activation energy *E_Mo_*, ascribed to a deep center *M*, and values of the photo-ionization cross-section *σ*_p_ have been extracted by using the Lucovsky approach, expressed as *σ*_p_ *= A*_MC_[*E*_Mo_^1/2^(h*ν* *−* *E*_Mo_)^3/2^]/(h*ν*)^3^. Here, h*ν* is the photon energy, and *A*_MC_ is a multiplicative factor dependent on parameters of the initial (*M*) and final (*C*) states. Excess carrier density *n*_ex_ generated by photo-ionization is determined by the absorption coefficient *α*(h*ν*) = *σ*_p_ (h*ν*) *n*_M_, which is proportional to a density of filled traps *n*_M_, and by the surface density of the incident photons *F*_ν_, as *n*_ex_ (h*ν*) = *α* (h*ν*) *F*_ν_ = *σ*_p_ (h*ν*) *n*_M_*F*_ν_ [14,20]. In a depleted diode base, the photo-generated excess carriers induce a current *i*, which is determined by carrier drift time *τ*_dr_ *= d*^2^/*µU*_R_ and, consequently, by parameters of the elementary charge *e*, of carrier mobility *µ*, of junction area *S* and of base thickness *d*, expressed as *i*(h*ν*) *= en*_ex_ (h*ν*) *SµU*_R_/*d*. Thereby, measured values of photo-induced current represent a step-like spectrum of carriers (and of *σ*_p_(h*ν*)) photo-excited from/to definite deep levels, as *F*_ν_ is kept constant. The spectral steps are simulated and assumed to be resolvable when amplitudes of the photoresponse signals differ about two times, like in the Rayleigh’s criterion for spectral lines.

## 4. Results and Discussion

The blocking junction of Cu_2_S-CdS for the majority carriers has been qualitatively tested by varying the polarity of the applied voltage and by measurements of pulsed barrier charging current transients induced by linearly increasing voltage (BELIV- [16,17]). The inherent shapes for the reverse biased BELIV transients (Figure 1) indicate that the high resistivity layer exists in the n-type conductivity CdS. The initial peak (Figure 1) of the barrier capacitance charging current is inherent for the reverse biased junction, followed by the descending current component due to the charge extraction determined reduction of the barrier capacitance when depletion width increases with voltage during a LIV pulse.

Three types of BELIV transients, associated with CdS polycrystal properties, have been obtained over the sets of Cu_2_S-CdS samples investigated in the dark. The square-wave shape BELIV transients are inherent for the I-type samples. These I-type BELIV transients imply the insulator-specific state of the base material, which lacks free carriers. For the II-type samples, the generation current component is pronounced within the rearward wing of the transient due to the carrier emission from deep traps. For the III-type samples, the barrier charging current peak prevails, which is accompanied with the descending current component due to charge extraction. For steady illuminated samples of type-II and type-III, barrier capacitance increases relatively to that value measured in the dark. This observation indicates a recovery of the junction under steady-state illumination. For the illuminated structure, the initial barrier capacitance charging current decreases with the enhancement of reverse voltage due to the widening of the depleted layer. However, the thermal emission current, which increases due to traps within the enhanced depleted volume, prevails within the rearward wing of the BELIV pulse. This indicates that all the investigated junction types exhibit a wide spectrum of defects acting as carrier capture/generation centers. Presence of a high density of carrier generation centers is additionally confirmed by differences in capacitance-voltage (C-V) characteristics. The observed differences over the BELIV transient shapes were explained [16,17], assuming variation of relative densities of dopants (which form shallow levels) and carrier capture centers (associated with deep levels). The value of the free carrier density of the *n*_0_= 1.5 × 10^13^ cm^−3^ is estimated for junctions of II- and III-type, and it determines concentration of shallow traps. For the I-type samples, a geometrical capacitance *C*_g_ can be only evaluated, which was found to be *C*_g_ = 3 nF, and this *C*_g_ value is in agreement with independently measured thickness *d* and probed area *S* of the CdS layer.

**Figure 2:**
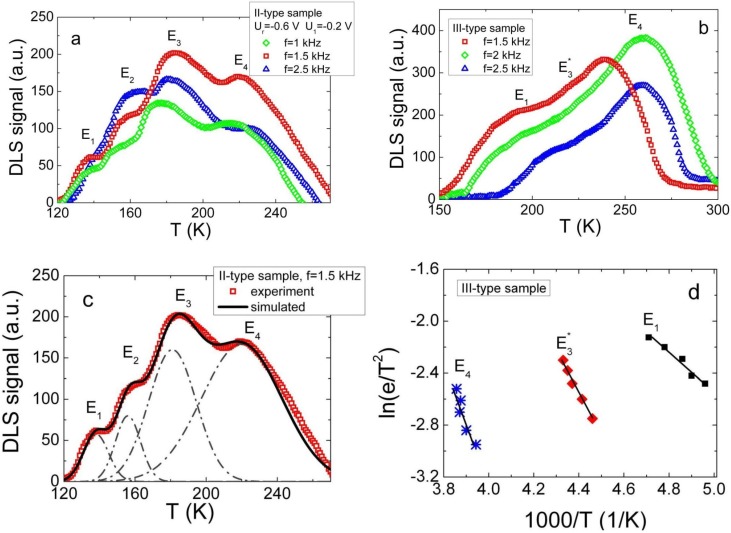
The capacitance deep level transient spectroscopy technique together with white LED light additional illumination (C-DLTS-WL) spectra obtained on samples of the II-type (**a**) and III-type (**b**) of Cu_2_S-CdS junction samples, as measured at 0.6 V reverse bias voltage using temperature scan regime for the lock-in frequencies range from 400 to 2500 Hz; (**c**) The simulated spectrum of sample of type-II; (**d**) The Arrhenius plots for III-type sample.

The intensity of the white LED was insufficient to get measurable DLTS signal on samples of I-type, containing the smallest equilibrium carrier density and exhibiting the capacitor-like BELIV and C-V characteristics. For the samples containing the II-type and the III-type junctions, the white light of LED illumination induces a stationary domain of the excess carriers, which make a virtual cathode and, thus, enables measurements of barrier capacitance changes due to the carrier thermal emission from deep levels. The C-DLTS-WL spectra obtained on samples of the II- and III-type are shown in Figure 2.

For the samples of II-type (Figure 2a), four overlapping peaks (Figure 2c) can be resolved. To identify prevailing traps, the experimentally measured spectra were simulated by Gaussian shape curves, like as in [21], to extract the DLTS signatures. It can be noticed that peaks at 138 K, at 157 K, at 183 K and at 225 K comprise a temperature scanned DLTS spectrum. The shifts of these peaks under varied lock-in amplifier frequency in the range of 1.5–2.5 kHz are in good agreement with predictable peak variations within routine DLTS spectroscopy. The range of DLTS signal filtering frequencies implies rather fast carrier emission times in samples of the II-type.

A more complicated DLTS-WL spectrum appears for samples of the III-type (Figure 2b). There, peaks associated with majority (at 205 K, at 232 K and at 255 K) carrier traps are present. A shift of these peaks with varied filtering frequency is also observable. However, thermal emission lifetimes in samples of the III-type are a little bit longer than those deduced from the peak shifts in samples of the II-type. The DLTS signatures are again extracted after the simulated peaks are fitted to the experimental ones for the majority carrier traps.

The Arrhenius plots (Figure 2d) using DLS-82E software have been made using the data obtained for variations of each peak temperature position ascribed to filtering frequency. Then, activation energy values have been extracted and listed in Table 1. Peaks from E_1_ to E_4_ were associated with electron traps, according to literature data [22,23,24,25,26,27] published for polycrystalline CdS. Also, DLTS peaks were analyzed using literature data for crystalline CdS and correlated with those for polycrystalline CdS.

**Table 1:** Deep level transient spectroscopy (DLTS) peaks obtained within the spectra of different types of samples.

Type of samples	Temperature at which DLTS peak is observed (K)	Energy of thermal activation of trap obtained from Arrhenius plot (eV)	Origin of the trap, reference
II-type	138	E_1_ = 0.15 ± 0.01	electrons-trap in polycrystalline CdS [23,26]
	157	E_2_ = 0.22 ± 0.02	electrons-trap in polycrystalline CdS [25,27]
	183	E_3_ = 0.26 ± 0.02	electrons-trap in polycrystalline CdS [26]
	225	E_4_ = 0.40 ± 0.02	electrons-trap in polycrystalline CdS [23]
III-type	205	E_1_ = 0.13 ± 0.01	electrons-trap in polycrystalline CdS [23]
	232	E_3_^*^ = 0.31 ± 0.02	electrons-trap in polycrystalline CdS [23,26]
	255	E_4_ = 0.4 ± 0.02	electrons-trap in polycrystalline CdS [23]

**Figure 3:**
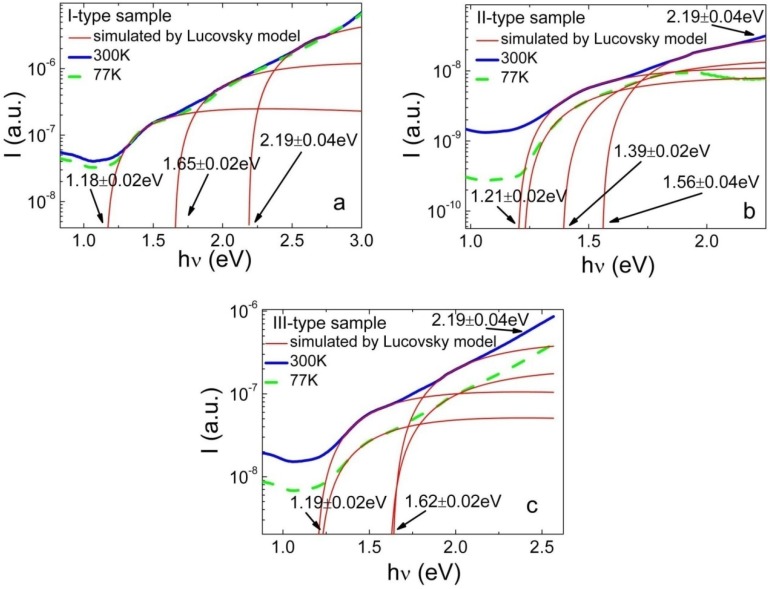
Photo-ionization spectra obtained on the I-type (**a**); on the II-type (**b**) and on the III-type (**c**) of Cu_2_S-CdS junction samples, as measured at 0.5 V bias voltage and at two different temperatures: at room (solid blue line), as well as at liquid nitrogen (dash green line). The thin red lines represent the simulated by Lucovsky model photo-ionization spectroscopy (PIS) steps fitted to the experimental spectrum.

The photo-ionization spectra measured for all the separated types of samples are shown in Figure 3. These spectra were recorded at room (solid blue line) and liquid nitrogen (dash green line) temperatures. It can be noticed that the BELIV separated types of samples are corroborated by different structure of the PIS spectra. Namely, the I-type junction samples contain the richest structure of traps with different photo-ionization activation energies (Figure 3a), and at least three steps can be easily distinguished using the Lucovsky approach. The II-type samples (Figure 3b) exhibit a two-three step structure of the measured PIS spectrum. Also, two-three PIS peaks can be resolved within the spectrum of the III-type samples (Figure 3c). The position of steps within each PIS spectrum is listed in Table 2. Activation energy values have been extracted on the basis of the fitted PIS steps by varying the red-threshold values within simulated electron-photon cross-section spectral variation curves. The obtained values of activation energy are listed in Table 2. The red-threshold activation energy values, extracted using the Lucovsky model, are also denoted in Figure 3.

**Table 2:** Photo-ionization spectroscopy (PIS) peaks obtained within spectra of different types of samples.

Type of samples	Photon energy at which PIS peak is observed (eV)	Energy of photo-activation of trap extracted by using Lucovsky approach (eV)
I-type	1.4	1.17 ± 0.02
	1.8	1.65 ± 0.02
	2.5	2.19 ±0.04
II-type	1.3	1.21 ± 0.02
	1.9–2.0	1.56 ± 0.04
	2.5	2.19 ± 0.04
III-type	1.3	1.19 ± 0.02
	2.0	1.62 ± 0.02
	2.5	2.19 ± 0.04

Leakage current, due to simultaneous thermal emission from different traps, increases a pedestal of the PIS signal significantly. Therefore, extraction of the activation energy for deep traps is more reliable using those PIS measured at 77 K temperature, while the existence of these peaks is clearly confirmed within PIS spectra measured at room temperature. Also, low temperature measurements are preferential, as an impact of the phonon-electron interactions are efficiently reduced with a decrease of temperature.

The combined analysis of the extracted values of DLTS and PIS peaks has been performed keeping in mind that DLTS directly represents thermal activation energy, while photo-ionization transitions are probable only from those filled to the empty levels. The correlated values of activation energies for thermal and photo-emission of carrier transitions are presented in Table 3.

**Table 3:** Correlation of PIS peaks with deep level transient spectroscopy (DLTS) peaks obtained within spectra of different types of samples.

Type of samples	Thermal activation energy (eV)	Energy of photo-activation of trap (eV)	Balance of activation energies for E_g_(300K) = 2.53 eV	Origin of the trap	References
I-type	–	1.17 ± 0.02	1.34 ± 0.02	electrons-trap, recombination center	[28]
	–	1.65 ± 0.02	0.90 ± 0.02	holes-trap, Cu impurities associated	[22]
	–	2.19 ± 0.04	0.34 ± 0.04	Cu impurities associated; electrons-trap in polycrystalline CdS	[22] [23,26]
II-type	–	1.21 ± 0.02	1.31 ± 0.02	electrons-trap, recombination center	[28]
	–	1.56 ± 0.04	1.0 ± 0.04	holes-trap, Cu impurities associated	[22]
	0.26–0.40	2.19 ± 0.04	0.34 ± 0.04	holes-trap, Cu impurities associated; electrons-trap in polycrystalline CdS	[22] [23,26]
III-type	–	1.19 ± 0.02	1.34 ± 0.02	electrons-trap, recombination centr	[28]
	–	1.62 ± 0.02	0.90 ± 0.02	holes-trap, Cu impurities associated	[22]
	0.31 ± 0.02	2.19 ± 0.04	0.34 ± 0.04	holes-trap, Cu impurities associated; electrons-trap in polycrystalline CdS	[22] [23,26]

Shallow levels identified by DLTS spectroscopy were not observable in PIS spectra, as PIS steps are hidden by absorption edge transitions for enhanced energy photons. The coincidence of DLTS and PIS activation energy has been obtained only for the level with an activation energy E = 0.34 ± 0.04 eV. A trap with such an activation energy is ascribed in the literature [22,23,24,25,26,27] to Cu impurities in CdS polycrystalline material. Deeper levels with an activation energy of 0.9 and 1.34 eV, identified by PIS measurements and observed in all the separated types of samples, can be associated with electron and hole traps, known as recombination centers in CdS crystals [28].

## 5. Summary

Three types of the Cu_2_S-CdS junction structures, separated by using the barrier capacitance characteristics of the junctions and correlated with XRD distinguished structural composition of the polycrystalline layers containing different Cu*_x_*S precipitates, exhibit different deep trap spectra within CdS substrates. Namely, the I-type junction samples, having the smallest free carrier densities and the largest concentration of traps, as deduced from BELIV transients, contain the richest structure of traps with different photo-ionization activation energies. The II-type and III-type samples exhibit the two-three step structure of the measured PIS spectrum. The C-DLTS-WL spectra can be recorded only in the samples of II- and III-type. There, only traps of the majority carriers have been distinguished in DLTS. The same level with activation energy E = 0.34 ± 0.04 eV has been identified by DLTS and PIS measurements. A trap with such an activation energy is ascribed to Cu impurities in CdS polycrystalline material. Deeper levels with an activation energy of 0.9 and 1.34 eV, identified by PIS measurements and observed in all the separated types of samples, can be associated with carrier traps, known as recombination centers in CdS material. The combined examination of the deep traps modified characteristics, by using barrier capacitance charging current, thermal- and photo-ionization spectroscopy techniques, appeared to be sufficient to separate the different sample types formed by slightly different layer deposition conditions.

## References

[1] Blachnik R., Müller A. (2000). The formation of Cu_2_S from the elements: I. Copper used in form of powders. Thermochim. Acta.

[2] Nan Z., Wang X.Y., Zhao Z. (2006). Formation of various morphologies of copper sulfides by a solvothermal method. J. Cryst. Growth.

[3] Grozdanov I., Najdoski M.J. (1995). Optical and electrical properties of copper sulfide films of variable composition. Solid State Chem..

[4] Banno N., Sakamoto T., Hasegawa T., Terabe K., Aono M. (2006). Effect of ion diffusion on switching voltage of solid-electrolyte nanometer switch. Jpn. J. Appl. Phys..

[5] Chopra K.L., Das S.R. (1983). Thin Film Solar Cell.

[6] Goldenblum A., Popovici G., Elena E., Oprea A., Nae C. (1986). All-evaporation-processed Cu_2_S/CdS solar cells with improved characteristics. Thin Solid Films.

[7] Aperathitis E., Scott C.G. (1989). The use of vacuum evaporation for production of the Cu_2_S absorber layer in thin-film CdS solar cells. J. Phys. Condens. Matter.

[8] Vanhoecke E., Burgelman M., Anaf L. (1986). Reactive sputtering of large-area Cu_2_S/CdS solar cells. Thin Solid Films.

[9] Soriano L., Leon M., Arjona F., Camarero E.G. (1985). On the photoconductivity of copper sulphide polycrystalline thin films. Sol. Energy Mater..

[10] Kundu M., Hasegawa T., Terabe K., Yamamoto K., Aono M. (2008). Structural studies of copper sulfide films: Effect of ambient atmosphere. Sci. Technol. Adv. Mater..

[11] Tang J., Huo Z., Brittman S., Gao H., Yang P. (2011). Solution-processed core–shell nanowires for efficient photovoltaic cells. Nat. Nanotechnol..

[12] Te Velde T.S. (1975). The production of the cadmium sulphide-copper sulphide solar cells. Energy Convers..

[13] Goldenblum A., Oprea A. (1994). Photocapacitance effects in dry processed Cu_2_S-CdS heterojunctions. J. Phys. D.

[14] Kalendra V., Bajarūnas D., Gaubas E. Comparative study of the photo-ionization and of DLTS characteristics in the hadrons irradiated Si detectors. Proceedings of Radiation Interaction with Material and Its Use in Technologies 2012.

[15] Martinuzzi S., Cabane-Brouty F., Gervais J., Mostavan A. Study of Cu2S-CdZnS photocells and CdS “spray” layers. II. First results on characterization of CdS spray layers. Proceedings of International Conference on Solar Electricity.

[16] Gaubas E., Čeponis T., Kalendra V., Kusakovskij J., Uleckas A. (2012). Barrier evaluation by linearly increasing voltage technique applied to Si solar cells and irradiated pin diodes. ISRN Mater. Sci..

[17] Gaubas E., Čeponis T., Sakalauskas S., Uleckas A., Velička A. (2011). Fluence dependent variations of barrier and generation currents in neutron and proton irradiated Si particle detectors. Lith. J. Phys..

[18] Blood P., Orton J.W. (1992). The Electrical Characterization of Semiconductors: Majority Carriers and Electron States.

[19] Lucovsky G. (1965). On the photoionization of deep impurity centers in semiconductors. Solid State Commun..

[20] Gaubas E., Uleckas A., Grigonis R., Sirutkaitis V., Vanhellemont J. (2008). Microwave probed photoconductivity spectroscopy of deep levels in Ni doped Ge. Appl. Phys. Lett..

[21] Gaubas E., Čeponis T., Uleckas A., Vaitkus J., Raisanen J. (2010). Recombination characteristics in 2–3 MeV protons irradiated FZ Si. Nucl. Instrum. Methods A.

[22] Poulin F., Brinkman A.W., Woods J. (1982). Electron and hole traps in the Cu*_x_*S-CdS heterojunction. J. Cryst. Growth.

[23] Besomi P., Wessels B. (1980). Deep level defects in polycrystalline cadmium sulphide. J. Appl. Phys..

[24] Hussein M., Lleti G., Sagnes G., Bastide G., Rouzeyre M. (1981). Deep level transient spectroscopy of electron traps and sensitizing centers in undoped CdS single crystals. J. Appl. Phys..

[25] Verity D., Shaw D., Bryant F.J., Scott C.G. (1983). DLTS investigation of electron traps in as-grown and Cd-fired CdS. Phys. Status Solidi.

[26] Claybourn M., Brinkman A.W., Russel G.J., Woods J. (1987). Electron traps in single-crystal CdS. Philos. Mag. B.

[27] Wang Z., Li B., Zheng X., Xie J., Huang Z., Liu C., Feng L.-H., Zheng J.-G. (2010). Deep level transient spectroscopy investigation of deep levels in CdS/CdTe thin film solar cells with Te:Cu back contact. Chin. Phys. B.

[28] Lashkarev V.E., Liubchenko A.V., Sheinkman M.K. (1981). Non-Equilibrium Processes in Photo-Conductors.

